# Parental satisfaction and perception of Progress in influencing the Practice of complementary health approaches in children with autism: a cross sectional survey from Negeri Sembilan, Malaysia

**DOI:** 10.1186/s12906-019-2672-8

**Published:** 2019-09-09

**Authors:** Jun Jean Ong

**Affiliations:** Pediatric Department, Clinical Campus Seremban, International Medical University (IMU), Jalan Rasah, 70300 Seremban, Seri Menanti, Negeri Sembilan Malaysia

**Keywords:** Autism spectrum disorder, Parent satisfaction, Complementary health approaches

## Abstract

**Background:**

Parents’ use of complementary health approaches (CHA) for children with autism spectrum disorder (ASD) are common despite the uncertain evidence of its benefit. Parents often adopt CHA due to dissatisfaction with conventional treatment. This study aimed to examine parents’ satisfaction with ASD treatment and their perception of progress in their child’s development. Parents’ use of CHA among children with ASD and the factors related were also evaluated.

**Methods:**

Self-administered questionnaires were completed by 48 parents of children with ASD at a single tertiary referral hospital in Malaysia. Correlation analysis was used to explore associations between parental satisfaction scores, perception of progress scores and use of CHA.

**Results:**

Use of CHA was reported by parents for 35.4% of children with ASD in the sample. Parents who were less satisfied with conventional treatment and parents who perceived poorer progress in their child’s development were more likely to use CHA. Strong positive relationship was found between parent satisfaction with ASD treatment scores and parent perception of progress scores, which indicates that parents who were satisfied with treatment were more likely to perceive greater progress in their child’s development. Improvement in child’s progress was most appreciated by parents in their child’s behavior (85.5%), social skills (83.3%) and motor skills (77.1%).

**Conclusion:**

The use of CHA was common among children with ASD. Parents were more likely to practice CHA when they were less satisfied with conventional treatment and perceived poorer progress. A larger multicenter study is required to further explore the practice of CHA among children with ASD throughout Malaysia.

## Background

Autism Spectrum Disorder (ASD) is a neurodevelopmental disorder characterized by persistent deficits in social communication and interaction across multiple context with restricted, repetitive patterns of behavior, interests or activities [1]. One of the main factors contributing to the rise of ASD prevalence worldwide is the growing awareness among health professionals. In Malaysia, the publication of the first local clinical practice guideline on the Management of ASD (2014) have resulted in increasing number of children suspected with ASD referred for early diagnosis and interventions [2].

Management of ASD requires a combination of regular behavioral and educational interventions that manages the main symptoms of autism, and occasionally medications to manage coexisting medical and behavioral problems. A high number of trained occupational therapist, speech therapist, psychologist and doctors specializing in ASD are required to address the rising number of children diagnosed. In Malaysia, all children with ASD receiving services from the public funded hospitals are facing challenges with the limited numbers of occupational and speech therapist available. Thus, it is fundamental to promote parental training and parent-mediated interventions to maximize the benefits of each treatment sessions [3]. The national clinical guidelines on Management of ASD equally emphasize on the importance of parental involvement to encourage continuity and application of learnt skills in the home environment. Parents has the advantage of recognizing changes early when monitoring treatment outcome due to their familiarity with their child’s uniqueness [4].

### Factors influencing parent satisfaction with ASD treatment

Parent satisfaction with treatment motivates parents to be actively involved in their child’s care. Their perception of treatment and perceived effectiveness are often taken into consideration by healthcare providers for decision making on treatment modalities and treatment goals. Parent satisfaction is also frequently used as an indicator of service quality [5], especially so in-service orientated healthcare system which will further influence evaluation, treatment outcome and quality of healthcare [6–8]. Additionally, parents assist in providing information about the child’s progress in functioning skills and behavior [4], therefore working alongside the therapist in deciding on treatment goals. Variability in characteristics and symptoms among children with ASD may also result in different treatment response. Treatment will most likely be discontinued if parents believe their child is not benefiting or making progress. Therefore, parents’ involvement and perception of child’s positive progress are essential to ensure adherence and strong parent- therapist alliance [9, 10].

### Services for ASD in Malaysia

Children with clinical suspicion of ASD or fails the Modified Checklist for Autism in Toddlers (M-CHAT) that is integrated in the health record book, are being referred to the state referral hospital. Diagnosis of ASD is made based on the DSM V criteria through information collected from different sources and clinical judgements of the pediatrician. Children diagnosed are subsequently managed with the occupational and speech language pathologist available in the state hospitals or health clinics [2]. The most common interventions implemented by occupational therapist in Malaysia are play therapy, followed by sensory integration training, sensorimotor stimulation, preschool training, early intervention and Snoezelen therapy [11]. The frequency of treatment received are often below international recommendation and depends largely on the availability of the therapist [12]. In addition, many occupational therapists may not have received specialized training or certification in providing therapy to children with ASD [11]. Hence, parents from middle and higher income may decide to send their child for additional regular private treatment which are costly and not supported financially by the national healthcare [13].

### Complementary health approaches (CHA) in Malaysia

Complementary medicine is defined by World Health Organization (WHO) as a broad set of health care practices that are not part of that country’s own tradition or conventional medicine and are not fully integrated into the dominant health-care system [14]. In Malaysia, the term is used interchangeably with traditional medicine which are practices based on the theories, beliefs, and experiences indigenous to different cultures [14]. Hence, for the purpose of this study, CHA refers to all complementary and traditional practices designed to prevent, treat or manage ailment or illness or preserve the mental and physical well-being of an individual which includes traditional Malay practices, traditional Chinese or Indian practices, Islamic medical practices, homeopathy and other complementary therapies [15].

Parents seek for CHA due to various reasons. Many are influenced by their culture and traditions, believes that CHA is a safe natural method and are swayed by the easy accessibility of the products in the community [16]. It had also been postulated that parents’ choice of CHA is based on their perceived cause of ASD [17, 18]. Unconfirmed efficacy of CHA spreads extensively through word of mouth and is easily accepted by parents who are searching for treatment that promises cure. A 2009 national survey involving all age groups had reported that CHA was practiced by more than half (55.6%) of the population to promote health, prevent and treat illness [19, 20]. Biological based therapies (88.9%) like herbs, supplements and vitamins are the most common CHA used followed by manipulative/ body-based practices (27%) used to treat health problems [19]. However, the use of CHA among children in Malaysia is still unclear especially among children with ASD. Little is also known about the linkage between parent satisfaction, perception of progress and the use of CHA for children with ASD in Malaysia. Therefore, this study aimed to examine parent satisfaction with ASD treatment and their perception of progress in their child’s development. Parents’ use of CHA among children with ASD and the factors related were also evaluated.

## Methods

Parents and caregivers of children below 18 years old diagnosed with ASD, who were attending the scheduled clinic appointment at Pediatric Clinic, Hospital Tuanku Jaafar were invited to participate. The hospital is a tertiary center that receives patient referrals from health clinics in the entire Negeri Sembilan state and its neighboring states in Malaysia. Newly diagnosed ASD within the last 6 months were excluded because it was felt that parents were not able to truly appreciate adequate progress in such a short time. During that period of time, the children may have attended two or three therapy session, which is too few for parents to build an objective opinion. Written informed consents were obtained and parents who agreed to participate were requested to complete a questionnaire. Questionnaires were mailed to parents of children who were not able to attend their appointment. A return stamped envelope was attached to encourage participation. Ethical approval was obtained from the Joint Committee of Research and Ethics of the International Medical University, Malaysia (IMU 389/2017) and Ethics Committee of National Malaysia Research Registry (NMRR-17-2521-37,205), prior to commencement of the study.

A total of 80 questionnaires were distributed. All of the 41 questionnaires distributed during the children’s pediatric clinic follow up were completed. However, 8 out of 39 mailed were returned (20.5% return rate). One questionnaire was not included due to incomplete data. The final completed questionnaires included in the study were 48.

### Study instrument

A self-administered questionnaire which consist of 4 sections covering the demographic data of both parent and child (section A), parent satisfaction with ASD treatment and parent perception of child’s progress (section B), practice of private conventional treatment or complementary health approaches (section C) and presence of comorbidities (section D) were distributed.

#### Demographics

Parents were requested to report their child’s age, gender, ethnicity, number of siblings and family income. Information on household family income were grouped into 3 categories which consist of low (< MYR3000.00, equivalent to USD730.00), middle (MYR3001.00–10,000.00, equivalent to USD730.00 – USD2400.00) and high (> MYR10000.00) income group. The range of income in each category were based on the Malaysian official statistic data [21]. The age when child was first suspected and diagnosed to have ASD, as well as the person who first raised the suspicion were also requested in this section.

#### Parent ASD treatment satisfaction

Parents were asked to indicate responses on a five point Likert scale (5 = Strongly Agree to 1 = Strongly Disagree) in response to the question stem ‘I am happy with the treatment received by my child at…..’ followed by the unit involved (occupational therapy, speech therapy and pediatric clinic) which are based in the same public hospital. All three question items scores were summed to obtain the parent satisfaction scores.

#### Treatment outcome

The items evaluating parent perception of child’s progress were adapted from previous studies [9, 22] Parents were asked to assess their child’s progress in 6 areas of development in response to treatment which includes speech and language, behavior, focus and attention, cognitive, physical and fine motor and social. Each area was defined for parents. The parents were asked to rate the progress on a 5-point Likert scale (5 = Strongly Agree to 1 = Strongly Disagree) in response to the question stem ‘I notice improvement in my child’s progress after treatment in the following areas….) followed by the developmental areas listed above. All six question items scores were summed to obtain the progress scores.

#### ASD services

Parents were asked if they were using CHA or have had used CHA in the past. Parents who did were than requested to list all treatment (not provided by the state hospital) they had practiced in the past and recently. A list of possible treatments, both private conventional treatments (e.g. occupational therapy, early intervention program, speech therapy) and CHA treatments (e.g. homeopathy, dietary supplements, massages, acupuncture, religious activities) were given to parents. The list was compiled from both local and overseas literature search to ensure completeness of the list [19, 20, 23]. Religious practices include prayers, faith healing and rituals that are practiced for the purpose of preserving health or treating illness.

#### Parent’s belief about cause of ASD

Parents were asked about their perception of the cause of ASD. The list of causes was adapted from previous studies and were later grouped into unknown/genetic cause, medical related causes and non-medical causes [17, 24, 25].

The questionnaire was piloted on 5 parents of children with ASD to ensure easy comprehension and appropriateness of the questions. Minimal adjustments to improved readibility were required.

### Statistical analysis

Data collected were analyzed using SPSS version 25. Descriptive statistics were used to describe the demographic data and parents’ responses, in terms of frequencies and percentages. The continuous variables which comprise of parent satisfaction scores and progress scores were analyzed by using Pearson correlation coefficient. Spearman’s correlation was used for the other variables that do not fulfil the key assumptions of parametric test.

## Results

The participants were 48 parents of 38 boys and 10 girls with ASD (Table 1). Their mean age were 6.1 years (SD = 2.1). Questionnaires were mainly completed by the mothers (*n* = 40). Parents were the first person to raise the suspicion of ASD in 63.5% of the children, followed by teachers (9.6%). Diagnosis of ASD were established in only 3 (6.3%) children before the age of 24 months. Genetic factor and unknown reasons were frequently identified as the cause of ASD (73.6%), followed by medical-related causes (7.5%) such as pregnancy-related complication and nonmedical causes (18.9%) which includes spiritual or magical reasons, excessive use of gadgets, vaccine and incorrect methods used in raising child.

**Table 1:** Characteristics of Parents and their Children with ASD

*N* = 48	n	%
Current Age (Range: 3.3–12.6 years)		
• < 24 months	0	0
• 2–5 years	16	33.3
• > 5 years	32	66.7
Race		
• Malay	34	70.8
• Chinese	9	18.8
• Indian	5	10.4
Age first suspected		
• < 24 months	8	16.7
• 2–5 years	35	72.9
• > 5 years	5	10.4
Age diagnosed		
• < 24 months	3	6.3
• 2–5 years	31	64.5
• > 5 years	14	29.2
*Person who first raised the suspicion		
• Parent	33	63.5
• Relative	4	7.7
• Teacher	5	9.6
• Family Friend	4	7.7
• Nurse	2	3.8
• Doctor	4	7.7
Siblings		
• Only Child	5	10.4
• 1 sibling	3	6.3
• > 1 siblings	40	83.3
Parent’s household income (RM)		
• Low (< 3000)	23	47.9
• Middle (3001–10,000)	19	39.6
• High (> 10,000)	3	6.3
Parent’s highest education level		
• Never attended formal education	0	0
• Primary school	1	2.1
• Secondary school	12	25.0
• College	14	29.2
• University	15	31.3
• Post graduate	6	12.5
Risk Factors		
• None	37	74.0
• Prematurity	2	4.0
• Family history of ASD	10	20.0
• Others	1	2.0
Body Mass Index (BMI)		
• Underweight	6	12.5
• Normal	34	70.8
• Overweight/Obese	8	16.7
Co-morbid Conditions*		
• None	21	42.0
• Global development delay/Intellectual disability	11	22.0
• Attention Deficit Hyperactivity Disorder (ADHD)	10	20.0
• Sleep issues	4	8.0
• Epilepsy	1	2.0
• Gastrointestinal issues	1	2.0
• Anxiety	1	2.0
• Others	1	2.0
Therapy at Private centers (*n* = 16)*		
• Early Intervention Programme/ Occupational therapy	15	31.3
• Applied Behavioral Analysis	1	2.1
• Tuition (One to one)	1	2.1
• Swimming class	1	2.1
• Art class	1	2.1
Parental Perception of the Causes of ASD*		
• Unknown/ Genetic	39	73.6
• Medical related causes	4	7.5
• Non-medical related causes	10	18.9
Number of Complementary Health Approaches Practiced		
• None	31	64.6
• 1	10	20.8
• 2 or more	7	14.6

*Some have more than one answer, thus the total will not add up to 100%

Majority of parents were from lower (47.9%) and middle (39.6%) income group. Correlational result indicate that lower family income was significantly associated with lower parental education level (r = .610, *p* < .0001). One third (33.3%) of children attended conventional therapy at private centers and were associated with higher family income (r = .325, *p* < 0.05). The types of private treatment reported were early intervention program, occupational therapy and applied behavioral analysis therapy.

### Relationship between parent satisfaction with ASD treatment and perception of Progress

The study found that most parents were highly satisfied with ASD treatment. 89.6 and 75% of parents rated a minimum good for both occupational and speech language therapy (Table 2). Parents’ perception of their child’s progress in different developmental areas were also positive, with the most progress seen in behavior (85.5%) followed by social skills (83.3%), physical and fine motor (77.1%), speech and language (75.1%), cognitive (74.9%) and focus and attention (68.7%) (Table 3). A large positive relationship between parent satisfaction with ASD treatment scores and progress scores were found, r = .699, *p* < .001 (Table 4). In addition, the results showed that family income was inversely associated with parent satisfaction with ASD treatment scores, (r = − .391, *p* < 0.01). Both parent satisfaction and progress scores were not significantly associated with gender, age diagnosed, parent education level and presence of comorbidities.

**Table 2:** Responses of parents in rating their satisfaction towards treatment received

Treatment	Very Good N (%)	Good N (%)	Unsure N (%)	Poor N (%)	Very Poor N (%)
Overall rating	17 (35.4)	23 (47.9)	5 (10.4)	3 (6.3)	0
Occupational therapy	24 (50.0)	19 (39.6)	2 (4.2)	1 (2.1)	2 (4.2)
Speech and language therapy	16 (33.3)	20 (41.7)	6 (12.5)	4 (8.3)	1 (2.1)

**Table 3:** Responses of parents in rating their child’s progress across developmental areas

Developmental Area	Very Good N (%)	Good N (%)	Unsure N (%)	Poor N (%)	Very Poor N (%)
Behavior	15 (31.3)	26 (54.2)	3 (6.3)	1 (2.1)	3 (6.3)
Social	12 (25.0)	28 (58.3)	4 (8.3)	2 (4.2)	2 (4.2)
Physical and fine motor	16 (33.3)	21 (43.8)	7 (14.6)	1 (2.1)	3 (6.3)
Speech and language**	15 (31.3)	21 (43.8)	5 (10.4)	3 (6.3)	3 (6.3)
Cognitive	13 (27.0)	23 (47.9)	7 (14.6)	2 (4.2)	3 (6.3)
Focus and Attention	10 (20.8)	23 (47.9)	11 (22.9)	2 (4.2)	2 (4.2)

* Arrange from highest to lowest progress

** May not achieve 100% due to missing data

**Table 4:** Correlation of parental satisfaction and child’s progress scores with related factors

	Satisfaction scores	Progress Scores	Family Income	Treatment at Private Centre	Use of CHA
Satisfaction scores	1	.699**	−.391*	−.097	−.345*
Progress Scores		1	−.158	−.082	−.356*
Family Income			1	.325*	.010
Treatment at Private Centre				1	.063
Use of CHA					1

* *p* < 0.05

** *p* < 0.001

### Relationship between complementary health approaches, parent satisfaction with ASD treatment and parent perception of Progress

**Fig. 1:**
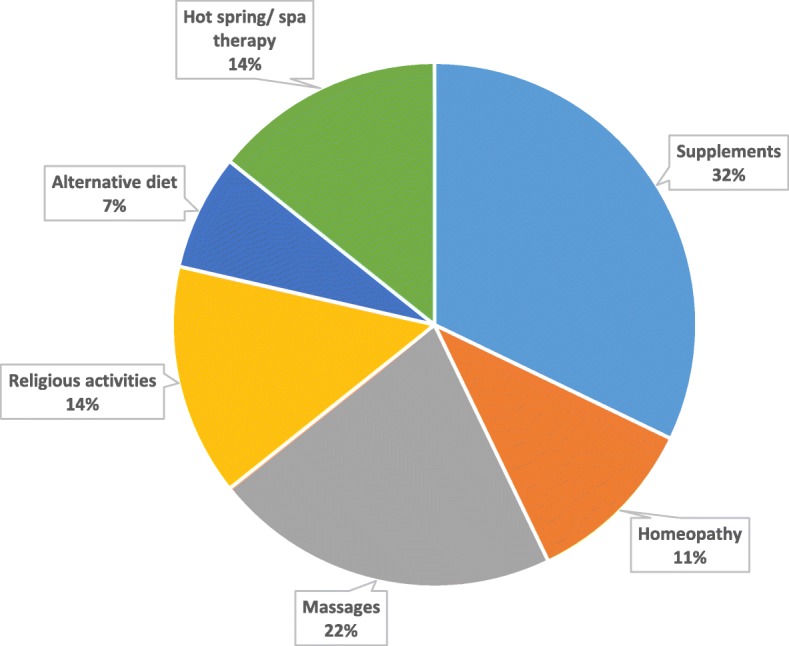
Complementary Health Approaches (CHA) Among Children with ASD

Practice of CHA were reported in 35.4%. The most common CHA were dietary supplements (32%), massages (22%), hot spring therapy (14%), and religious activities (14%) (Fig. 1). Correlational results indicated that there is a negative association between the use of CHA with parent satisfaction with ASD treatment scores and progress scores, (r = −.345, *p* < 0.05) and (r = −.356, *p* < 0.05) respectively. There was no association between parents’ perception of the cause of ASD and the use of CHA.

## Discussion

The study revealed that parents’ perception of their child’s progress in different areas of development were highly associated with parents’ satisfaction with ASD conventional treatment. All parents hope to achieve meaningful and functional improvement in their child’s behavior and development. Parents regard their child’s progress as a reflection of treatment effectiveness. Therefore, regardless of the child’s performance, if the parent’s perception of progress is not aligned with the healthcare providers and considers treatment as ineffective, the parent will not be satisfied with the treatment provided. At times, parents and healthcare providers may have different views on the amount of progress expected and disagree on treatment plans. These differences exist because the degree of improvement is subjective and may be influence by beliefs and practices [23, 26]. Perhaps parents’ view their child’s development at a different standpoint or reflects different priorities set among parents. However dissimilar the perceptions, it is still important to understand these differences because it will influence the parents’ future decision surrounding treatment [9, 27]. Lack of parents’ perception of change and improvement are reported as common reasons parents discontinue treatment [9, 28]. Therefore, shared decision making between both parents and healthcare providers, with consideration of parents’ priorities, are vital to discuss mutual realistic treatment goals to prevent noncompliance and disheartenment [29].

In the present study, parents were less satisfied with speech language therapy, compared to the occupational therapy. The disparity may be due to infrequent number of sessions secondary to the lack of trained speech language pathologist in the country [30]. It had been reported in a local study that speech therapy is among the third highest service that is very much needed and highly unmet among children with disabilities [31].

Use of CHA among children with ASD in this study (35.4%) were lower than the national statistics of 55.6% [19]. Previous local studies reported a higher use of CHA of 84.5% among children with cancer and 76% among children with inflammatory bowel disease [32, 33]. Perhaps parents of ASD children were more cautious when using CHA compared to parents of children with the above diseases. A small minority of parents may also be concern of the potential harm of the use of CHA [34] and only consider the option when truly necessary.

This study further showed that parents who perceived poorer progress in their child’s development and less satisfied with conventional treatment were more likely to use CHA. This result is consistent with findings from other studies [20, 35]. Parents are more likely to seek for treatment options like CHA that is easily accessible, cheaper and accepted by the community [35]. The use of CHA is further promoted by the perception that CHA is a safer ‘natural’ method that treats the cause, not just the symptoms of ASD and promotes resolution in core symptoms of ASD [18, 35, 36]. The commonest types of CHA used in this study were herbal and vitamin supplements, which were similar to practices in the local and Western population [35]. Religious activities were practiced by 14% of parents, however the nature of the religious activities were not explored in this study and may be underreported as a treatment method due to its integration into daily religious practices. A local study reported on the frequent use of spiritual therapy among Malaysian population, which comprise of faith healing or prayer that is strongly influenced by religion and culture [20]. According to the national policy guidelines on traditional and complementary medicine, spiritual therapies practiced for the purpose of restoring or maintaining health are recognized as a type of traditional medicine [37]. Homeopathy is a common practice among the Malay ethnic group in Malaysia, however it was reported to be surprisingly low in this study. The use of homeopathy was consistently found to be low in another local study among Malaysian population, and it was postulated that majority of participants may not have described the type of CHA used accurately thus wrongly categorized [19].

An association between parental beliefs on the cause of autism and the choice of CHA were not appreciated in this study [17]. This is perhaps due to the parents’ willingness to try all forms of treatment within their means regardless of their own beliefs. Additional factors that may have influence their choice of treatment were not explored here. Parents may also be pressured to use CHA due to the lack of established treatment options, high cost of conventional therapy and lack of transportation [38]. It had been reported that the cost of therapy at private occupational therapy centers may be as high as MYR 600 (estimated USD 150) per month or higher if more sessions are required, thus limiting the number of families that are able to afford private conventional treatment [13]. Although the cost of CHA may be high too, the range varies widely depending on the type of product or intervention and qualification of the practitioner, which allows more flexibility among parents of all income groups.

The small sample size was a limitation to this study. However, this is an exploratory study that may spur future research to investigate the factors influencing outcome of children in response to ASD treatment and the effectiveness of specific CHA in children with ASD. This study represented mainly middle and lower income group families and may not reflect children who were attending private medical centers. In addition, parents who were non-compliant to treatment and perhaps less satisfied with ASD treatment were also underrepresented as indicated by the poor return rate of the mailed questionnaires. The possibility of response bias among the parents who participated during their clinic appointments cannot be totally ignored and may be reduced by mailing all questionnaires to all participants. However, it was not implemented due to the predicted lack of response. Healthcare providers were also encouraged to enquire about the use of CHA and to allow parents to speak freely without feeling the need to hide the information.

## Conclusion

This study is the first to investigate parents’ satisfaction and perception of progress in children with ASD in Malaysia. The findings of its strong association further reinforce the importance of selecting therapy that focuses on parents’ outcome goals and interventions that highlights a particular target skill. A future systematic qualitative research will enlighten us on additional variables that may influence parental satisfaction. In addition, the practice of CHA is common among children with ASD in our center. However, this finding should be further ascertain by future studies exploring the actual prevalence of CHA among children with ASD at national level and identify additional contributory factors that leads to the adoption of CHA.

## Data Availability

The datasets used and analyzed during the current study are available from the corresponding author on reasonable request.

## Abbreviations

ASD: Autism Spectrum Disorder
CHA: Complementary Health Approaches
M-CHAT: Modified Checklist for Autism in Toddlers
